# Mass spectrometric insights into the protein composition of human cutaneous neurofibromas: comparison of neurofibromas with the overlying skin

**DOI:** 10.1038/s41416-025-03055-9

**Published:** 2025-05-20

**Authors:** Roope A. Kallionpää, Eija Martikkala, Pekka Haapaniemi, Sanna-Maria Karppinen, Pilvi Riihilä, Anne Rokka, Ilmo Leivo, Taina Pihlajaniemi, Sirkku Peltonen, Juha Peltonen

**Affiliations:** 1 Cancer Research Unit, Institute of Biomedicine, University of Turku, Turku, Finland; 2 FICAN West Cancer Centre, University of Turku and Turku University Hospital, Turku, Finland; 3 Turku Bioscience Centre, University of Turku and Åbo Akademi University, Turku, Finland; 4 ECM-Hypoxia Research Unit, Faculty of Biochemistry and Molecular Medicine, University of Oulu, Oulu, Finland; 5 Department of Dermatology and Venereology, University of Turku, Turku, Finland; 6 Department of Dermatology, Turku University Hospital, Turku, Finland; 7 FICAN West Cancer Research Laboratory, University of Turku and Turku University Hospital, Turku, Finland; 8 Department of Dermatology and Venereology, Institute of Clinical Sciences, Sahlgrenska Academy, University of Gothenburg, Gothenburg, Sweden; 9 Department of Dermatology and Venereology, Region Västra Götaland, Sahlgrenska University Hospital, Gothenburg, Sweden; 10 Department of Dermatology and Allergology, University of Helsinki, Helsinki, Finland; 11 Inflammation Center, Helsinki University Hospital, Helsinki, Finland

**Keywords:** Tumour-suppressor proteins, Oncogenesis, Tumour angiogenesis, Cancer microenvironment, Mechanisms of disease

## Abstract

**Background:**

Cutaneous neurofibromas (cNFs) are the hallmark of the tumor-predisposition syndrome neurofibromatosis 1 (NF1). While cNFs are always benign, they markedly decrease quality of life in individuals with NF1. Understanding the differences between cNFs and the skin is essential for developing treatments for cNFs.

**Methods:**

We collected 15 cNFs from four NF1 individuals and used mass spectrometry to compare the tumor tissue with the skin overlying each tumor. Data were analyzed based on Gene Ontology (GO) terms.

**Results:**

The expression patterns of the Schwann cell marker S100B and several keratins confirmed successful dissection of cNF tissue from the overlying skin. Hierarchical clustering showed extensive overlap between the tumor and skin samples in three out of four individuals, suggesting high overall similarity between the two tissue types. Based on the analysis of the GO terms, cNFs were associated with decreased expression of proteins related to cell proliferation, extracellular matrix remodeling, angiogenesis and cellular metabolism.

**Conclusion:**

The cNFs are relatively quiescent, consistent with their benign nature and limited growth potential. The development of pharmacological therapy for cNFs requires overcoming the high similarity between cNFs and the overlying skin. The present dataset can serve as a resource for future research on cNFs.

## Introduction

Cutaneous neurofibromas (cNFs) are the characteristic tumors of neurofibromatosis type 1 (NF1) [1, 2]. The cNFs typically start to grow during puberty, their number increases with age [3–6], and they are present in the majority of adults with NF1 [3, 4, 7]. These benign tumors never turn malignant, and they cease growth after reaching a maximum size of a few centimeters [2, 8–10], yet individuals with NF1 may have hundreds or even thousands of these tumors on their skin [3]. The cNFs can markedly decrease the quality of life through pain, itching and disfigurement [11–14]. Currently, the treatment of cNFs is almost exclusively based on surgical or laser excision [15], yet pharmacological treatments are being investigated [16]. Due to the benign nature of cNFs, major adverse effects of the treatments are unacceptable, prompting interest in topically administered drugs [16].

The NF1 syndrome is caused by pathogenic germline variants of the *NF1* tumor suppressor gene in chromosome 17 [17, 18]. With a prevalence of 1/3000 to 1/2000, NF1 is one of the most common genodermatoses [19, 20]. In addition to cNFs, NF1 is associated with plexiform neurofibromas (pNFs) [1]. In contrast to cNFs, pNFs can weigh several kilograms, cause pain and functional deficits, and undergo transformation to malignant peripheral nerve sheath tumor (MPNST) [1, 21]. Other manifestations characteristic of NF1 include café-au-lait macules, skinfold freckling, iris Lisch nodules, optic pathway gliomas and skeletal defects [22]. NF1 follows an autosomal dominant trait and shows full penetrance [19]. Approximately half of the affected individuals have inherited the disorder, while the other half have a de novo pathogenic variant [19, 23]. In addition to cNFs, pNFs and MPNSTs, NF1 is associated with an increased risk for many cancers including those of the brain, breast and gastrointestinal tract [24, 25].

The cNFs lie within the dermis, yet they are distinct from the surrounding skin. The cNFs always feature a clonal subpopulation of Schwann cells with somatic inactivation of the healthy *NF1* allele [26, 27]. In addition to Schwann cells, cNFs contain fibroblasts, perineurial cells, mast cells, macrophages and lymphocytes, and an abundant collagenous matrix [28–33]. All cell types of the peripheral nerve contribute to the accumulation of extracellular matrix in cNFs. The tumor matrix contains fibrillar collagen types I, III, and V, as well as fibronectin and proteoglycans [28]. The cNF Schwann cells and perineurial cells are surrounded by basement membranes, and basement membrane components such as laminin chains are also present in cNFs [28]. In addition, Schwann cells, perineurial cells and fibroblasts express genes for collagen VI [34, 35]. Experiments performed in murine models of pNFs have demonstrated a rich network of interactions between Schwann cells and the neurofibroma microenvironment [36–40]. While often considered highly vascularized, the vessel density of cNFs is lower than that seen in MPNSTs [41].

The present study aims to provide a characterization of the differences between cNFs and the overlying skin using mass spectrometry. The resulting dataset is a valuable resource for studying differences and similarities between cNFs and the surrounding skin. The development of topical treatments for cNFs highlights the need to identify these similarities and differences between the two tissue types to optimize anti-tumor efficacy and to avoid adverse effects.

## Materials and methods

### Patients and samples

The study was approved by the Ethics Committee of the Hospital District of Southwest Finland, and had a research permit from the Turku University Hospital. The study adhered to the principles of the Declaration of Helsinki. Written informed consent was obtained from the participants. The tumor samples were obtained from patients visiting the NF1 clinic operative in Turku University Hospital, Turku, Finland. All sample donors fulfilled the diagnostic criteria for NF1 [22, 42].

The cNFs for mass spectrometry were 3–10 mm in diameter, and they were excised using CO_2_ laser upon the patient’s initiative. The overlying skin was immediately dissected apart from the cNF, and both tissues were snap frozen and stored at −80 °C. Altogether 15 tumors from one male and three females were included in the study, with 3–5 tumors per patient. Eight tumors from two patients were estimated to be growing based on the report given by the patients, whereas there was no evidence of recent growth for seven tumors. The patients were aged 28–74 years at tumor excision, with a mean age of 48.6 years and a median of 40.7 years.

For the validation of the mass spectrometry results, 20 cNFs from seven individuals with NF1 (four males and three females) were excised using CO_2_ laser, formalin-fixed, paraffin-embedded and cut into 3 µm sections for immunohistochemistry.

### Sample preprocessing

Tissue pieces of 2 × 2 × 2 mm were sonicated for five cycles of 30 s on, 30 s off in 50 µL of 100 mM triethylammonium bicarbonate buffer supplemented with Complete Mini protease inhibitors (Roche, Basel, Switzerland). Pressure-based cell lysis was performed for 60 cycles of 50 s at 30 °C and 310 MPa using the Barocycler NEP 2320 instrument (Pressure BioSciences, Easton, MA, USA). The samples were sonicated for five cycles of 30 s on, 30 s off to disrupt DNA. After adding sodium dodecyl sulfate to a final concentration of 5%, the samples were incubated at 95 °C for 5 min and cooled to room temperature. The samples were centrifuged at 17,000 g for 20 min and the non-soluble material was discarded.

Protein concentration was determined using the Lowry method, and 50 µg of protein was used for trypsin digestion. For the reduction of the proteins, dithiothreitol was added to a final concentration of 20 mM, and the samples were incubated at 37 °C for 1 h. The proteins were then alkylated by adding iodoacetamide to a final concentration of 40 mM and incubation at room temperature for 30 min. After acidification with phosphoric acid and addition of the S-Trap binding buffer, the samples were applied to S-Trap micro spin columns (ProtiFi, Farmingdale, NY, USA). After washing, 2 µg of trypsin was added in 20 µL of 50 mM ammonium bicarbonate buffer, followed by incubation at 37 °C for 1 h, and at 47 °C for 1 h. The resulting peptides were eluted from the column using 40 µL of a 1:1 mixture of 50 mM ammonium bicarbonate buffer with 0.2% formic acid, and acetonitrile with 0.2% formic acid. The peptides were evaporated to dryness.

### Mass spectrometry

A nanoflow high-performance liquid chromatography system Easy-nLC 1200 coupled to the Q Exactive HF mass spectrometer (Thermo Fisher Scientific, Bremen, Germany) equipped with a nano-electrospray ionization source was used for liquid chromatography-electrospray ionization-MS/MS analyses. A total 600 ng of peptides were first loaded on a trapping column and subsequently separated inline on a 15-cm C18 column (75 μm × 15 cm, ReproSil-Pur 3 μm 120 Å C18-AQ; Dr. Maisch HPLC GmbH, Ammerbuch-Entringen, Germany). The flow rate was 300 nL/min. The mobile phase was solvent A: water with 0.1% formic acid; and solvent B: acetonitrile/water 80:20 (v/v) with 0.1% formic acid. A two-step gradient consisted of 90 min from 5% to 23% of solvent B, followed by 30 min from 23% to 36% of solvent B. Data-independent acquisition (DIA) method was used, and the duty cycle contained one full scan (400–1000 m/z) and 40 MS/MS scans covering the mass range 400–1000 with an isolation window of 15 m/z. The data were acquired automatically using Thermo Xcalibur 4.1 software (Thermo Fisher Scientific) and processed with the Spectronaut Pulsar software (Biognosys, Schlieren, Switzerland). Pulsar is a search engine, integrated into Spectronaut for spectral library generation. A spectral library containing data from all DIA runs was generated with Pulsar, and proteins from individual samples were then identified by comparison against the generated spectral library. The raw dataset covered 3002 different proteins. Data were normalized before analysis.

### Data processing

To exclude low confidence protein identifications, we required for each protein *P* value <0.05 and identification based on at least two peptides in at least one sample. Application of these criteria led to the inclusion of 2461 proteins in the final dataset. The proportion of missing proteins was 0.9–6.4% per sample. Among the cNF samples, an average of 99.4% of proteins (range 99.2% to 99.6%) were observed in at least one sample of a donor, and 93.0% (range 92.2% to 94.4%) in all samples of a donor. The respective numbers were 99.7% (range 99.3% to 99.9%) and 95.1% (range 91.6% to 97.7%) in the skin samples. Protein identification was based on a median of 5 peptides (range 1 to 454) and a median sequence coverage of 19% (range 0.1% to 100%).

Any remaining missing values in the dataset were either missing at random due to random failure to detect the protein, unrelated to the nature of the respective peptides or their measured intensity; or missing not at random because of expression level below the limit of detection [43]. Since missing values caused by low protein expression are informative, they were imputed with the minimum protein quantity value observed in the dataset. We assumed that a maximum of 20% of observations could be missing at random. If a protein could be quantified in ≥80% of samples of one tissue type (cNF or skin), the detection was considered reliable and any missing values in excess of 20% in the other sample type were assumed to be due to an expression level below the limit of detection and therefore imputed. If the protein was detected in less than 80% of samples in both tissue types, no imputation was performed to avoid imputing missing values caused by protein lability or technical issues. As a result, we may have missed proteins whose expression is low in both skin and cNF, yet these were not of our primary interest, as we aimed to characterize proteins differentially expressed in cNF and skin. Application of this procedure led to the imputation of 472 missing values (0.64% of all measurements; 373 values in cNF samples and 99 values in skin samples) in 119 proteins (4.8%). After the imputation, 99.96% of the 2461 proteins had data in both cNF and skin. A total of 1430 missing values in 499 proteins remained, and these were assumed to be missing at random.

### Statistical analysis

The proteins detected in the samples were linked to Gene Ontology (GO) terms (release 2021-07-02) [44, 45], and the GO terms associated with more than four different proteins in the dataset were analyzed. The analysis focused particularly on the GO terms related to extracellular matrix, angiogenesis and cellular metabolism. The cNF and skin were compared using linear mixed effects regression modeling of the log-transformed protein quantities, and a random intercept for each protein and nested random intercepts for each patient and sample were included. In case of model non-convergence, the models were simplified by first omitting the random intercept for each patient and then, if necessary, the random intercept for each sample. In addition, keratins, collagens and the Schwann cell marker S100 calcium-binding protein B (S100B) were analyzed at the level of individual proteins to confirm the successful dissection of the cNF from the overlying skin and to further describe the differences between cNFs and the skin. The single-protein analyses were performed similarly to the analyses of the GO terms, yet the random intercept for each protein was not included. The *P* values were Bonferroni corrected to account for the multiple comparisons. Sensitivity analyses encompassing specific subsets of tumors were used to validate the robustness of the results.

Correlations between GO terms were assessed using linear mixed effects regression with a random intercept for each patient. In order to do this, the quantity of each protein related to a specific GO term in each sample was normalized relative to the average quantity of the protein observed over all samples. The normalized protein quantities were averaged over the different proteins to obtain a sample-specific index for each process of interest, and log-transformed values were analyzed. The GO terms for angiogenesis (GO:0001525), cell population proliferation (GO:0008283), and positive regulation of mitogen-activated protein kinase (MAPK) cascade (GO:0043410) were correlated with the GO terms for extracellular matrix disassembly (GO:0022617), cellular protein metabolic process (GO:0044267), carbohydrate metabolic process (GO:0005975), and lipid metabolic process (GO:0006629).

To describe the overall similarity between cNFs and the skin, protein-level data were visualized as heatmaps and hierarchical clustering was performed using the complete linkage method. All analyses were performed using the R software for statistical computing, version 4.0.0, and package lmerTest, version 3.1-2. *P* values <0.05 were considered statistically significant.

### Immunohistochemistry

In order to validate the findings from mass spectrometry, 20 samples were labeled for the cell proliferation marker Ki67 and collagens XV and XVIII. The Ki67 immunohistochemistry was performed using the BenchMark Ultra system (Roche). A 36-min pretreatment with the Cell Conditioning Solution CC1 (Roche) was followed by a 12-min incubation with the CONFIRM anti-Ki67 (clone 30-9) rabbit monoclonal primary antibody (Roche) and detection with the UltraView Universal DAB detection kit (Roche). For collagen XV and XVIII immunostainings, epitope retrieval was performed using a heat-mediated method for 15 min with Tris/EDTA buffer (pH 9), followed by an incubation with polyclonal anti-collagen XV (Sigma-Aldrich, HPA017913, 1:1000) or in-house monoclonal anti-collagen XVIII (ref. [46], DB144-N2, 7 µg/ml) primary antibody. The labeling was carried out using the peroxidase-based Dako EnVision kit (Agilent Dako, Santa Clara, CA, USA) according to the manufacturer’s protocol. For negative controls, the primary antibodies were omitted and replaced with phosphate-buffered saline. All sections were counterstained with hematoxylin and imaged using the NanoZoomer S60 digital slide scanner (Hamamatsu, Hamamatsu City, Japan) and Pannoramic Midi FL slide scanner (3DHistech Ltd, Budapest, Hungary).

## Results

We performed mass spectrometry analysis of 15 cNFs and matched samples of the overlying skin from four individuals with NF1. The successful dissection of the cNF tissue from the overlying skin was confirmed by the 2.21-fold (95% CI 1.69 to 2.88, *P* < 0.001) expression of the Schwann cell marker S100B in cNFs compared to skin. Moreover, the expression levels of most keratins were <10% in cNFs compared to skin, and the expression of collagen XVII, characteristic of the epidermal-dermal junction, was very low in cNFs.

**Fig. 1:**
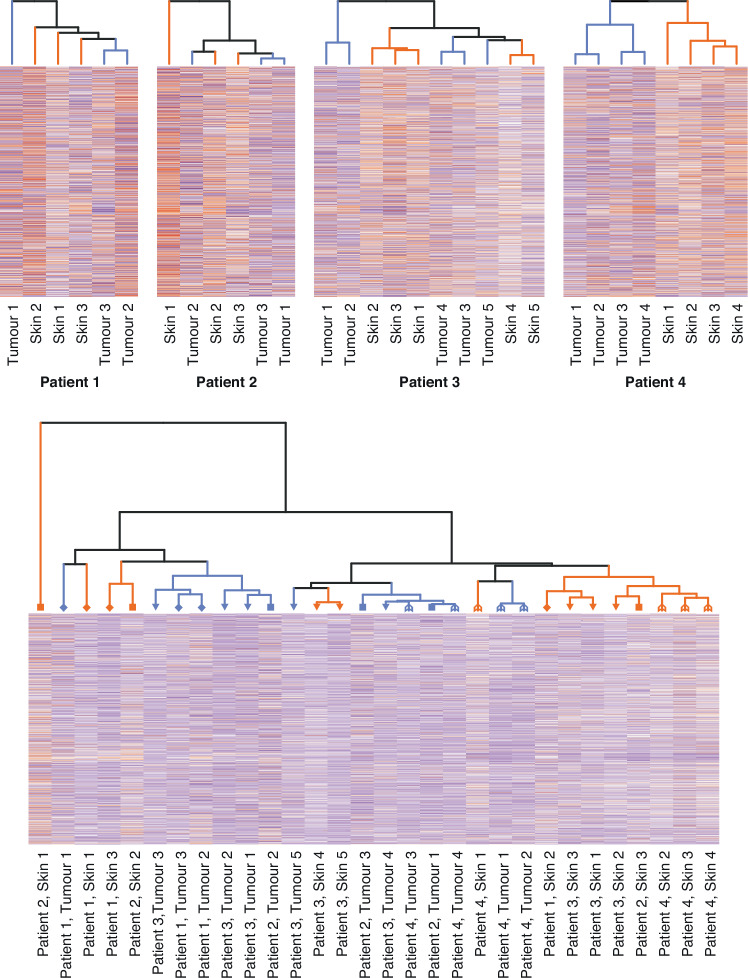
Hierarchical clustering of cutaneous neurofibromas and the overlying skin based on protein quantities. The top row shows results of individual-wise clustering, and the bottom panel displays pooled clustering of all the samples. In the dendrograms, tumor samples are highlighted in blue, and skin samples in orange. In the lower panel, each individual is denoted with a unique symbol in the dendrogram.

In one of the four sample donors (patient 4), complete separation of the cNF samples from the matched skin samples was seen in a hierarchical clustering analysis. The samples from the other three individuals showed overlap between the cNF and skin: the overall protein content of some cNF samples was closer to skin than to other cNFs (Fig. 1). When the samples from all individuals were pooled into a single analysis, subclusters based on tissue type rather than patient emerged (Fig. 1), indicating tissue-specific patterns of protein expression shared by multiple individuals. However, the separation was incomplete.

**Fig. 2:**
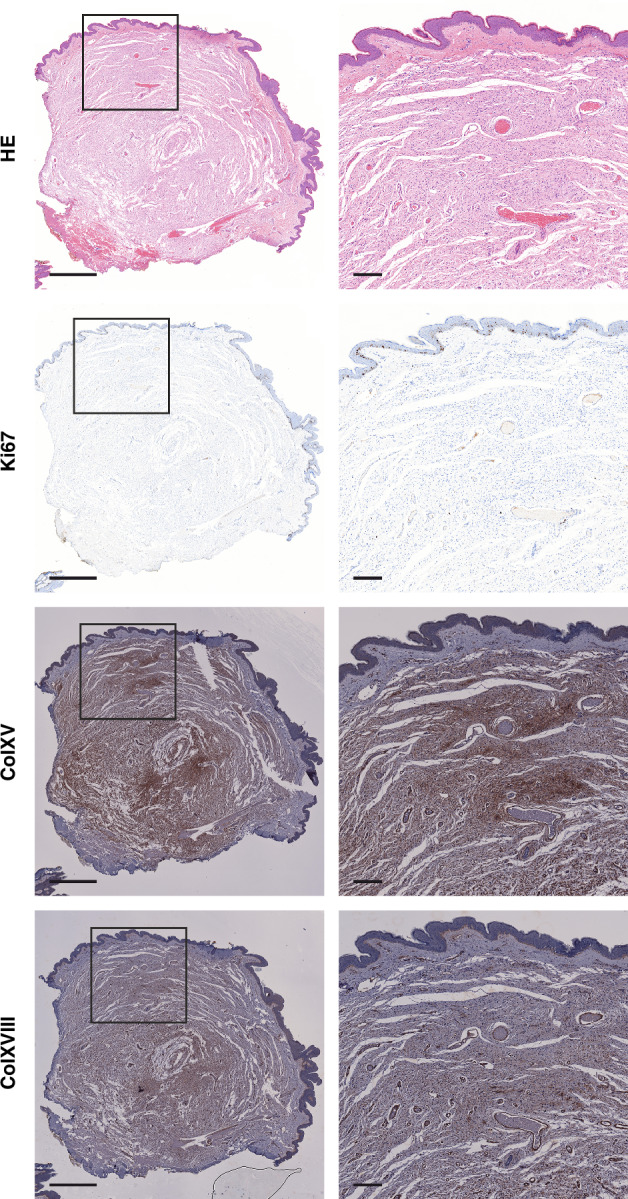
Hematoxylin-eosin staining and immunohistochemical labeling for the cell proliferation marker Ki67, and collagens XV (ColXV) and XVIII (ColXVIII) of a representative cutaneous neurofibroma. A total of 20 cutaneous neurofibromas from seven NF1 patients were examined. Proliferating cells are sparse within the tumor compared with the overlying skin whereas ColXV and ColXVIII are predominantly expressed in the tumor tissue. Scale bars 1 mm in the first column, 200 µm in the second column.

Based on data from 22 different proteins, the GO term for cell population proliferation (GO:0008283) was associated with an 11% (95% CI 3.2% to 18%, *P* = 0.006) lower level in cNFs compared to skin. Patient 4 was the only patient whose samples showed complete separation of cNFs and matched skin in the hierarchical clustering analysis. A sensitivity analysis encompassing only samples from this patient displayed a highly concordant 15% (95% CI 2.5% to 26%, *P* = 0.022) lower level of protein expression related to cell proliferation in cNFs vs. the skin. When the analysis was limited to presumably growing tumors only, the reduction in protein expression related to cell proliferation was 7% (95% CI −4% to 17%, *P* = 0.221) in cNFs vs. the skin, which was no longer significant. The low rate of cell proliferation in cNFs was confirmed by the Ki67 immunolabeling of 20 cNFs. Few Ki67 positive cells were observed within cNFs, which is in contrast to the numerous proliferating cells in the epidermis (Fig. 2). The positive regulation of MAPK cascade (GO:0043410) did not differ between cNFs and the overlying skin in all tumors (0.93, 95% CI 0.77 to 1.13, *P* = 0.481; 15 proteins), nor in growing tumors only (0.93, 95% CI 0.69 to 1.24, *P* = 0.619). However, the samples from patient 4 displayed a statistically significant decrease (0.83, 95% CI 0.71 to 0.98, *P* = 0.026).

### Extracellular matrix

Cutaneous neurofibromas are known to harbor an abundant collagenous matrix [28, 29]. Consistent with this, extracellular matrix binding (GO:0050840) was associated with a 110% (95% CI 52% to 189%) higher level in cNFs compared to the overlying skin (Table 1). In addition, the expression of proteins related to extracellular matrix structural constituents (GO:0005201) was 25% (95% CI 11% to 41%) higher in cNFs. The expression level of proteins related to basement membrane organization (GO:0071711) was 109% (95% CI 51% to 189%) higher in cNFs compared to the skin, yet the expression of proteins related to basement membrane as a whole (GO:0005604) did not differ between the two tissues. The proteins related to the GO term for collagen binding (GO:0005518) were 13% (95% CI 0% to 27%) more abundant in cNFs than in the skin, yet the difference was not significant after correction for multiple comparisons (Table 1). The estimates were highly concordant in sensitivity analyses including growing tumors only, or patient 4 only (Supplementary Table 1).

**Table 1:** A comparison of Gene Ontology (GO) terms related to extracellular matrix in cutaneous neurofibromas vs. the overlying skin.

GO term	Proteins	Estimate (95% CI)	*P*_*plain*_	*P*_corrected_^a^
Extracellular matrix disassembly [GO:0022617]	25	0.41 (0.32 to 0.52)	<0.001	<0.001
Collagen biosynthetic process [GO:0032964]	5	0.60 (0.47 to 0.76)	<0.001	0.002
Extracellular space [GO:0005615]	454	0.69 (0.66 to 0.72)	<0.001	<0.001
Mitochondrial matrix [GO:0005759]	92	0.76 (0.73 to 0.79)	<0.001	<0.001
Extracellular region [GO:0005576]	554	0.83 (0.80 to 0.85)	<0.001	<0.001
Collagen trimer [GO:0005581]	20	0.84 (0.71 to 0.99)	0.041	1.000
Collagen catabolic process [GO:0030574]	10	0.87 (0.76 to 0.99)	0.040	1.000
Collagen binding [GO:0005518]	31	1.13 (1.00 to 1.27)	0.047	1.000
Cell-matrix adhesion [GO:0007160]	49	1.15 (1.01 to 1.31)	0.036	1.000
Extracellular matrix structural constituent [GO:0005201]	51	1.25 (1.11 to 1.41)	<0.001	0.010
Basement membrane organization [GO:0071711]	8	2.09 (1.51 to 2.89)	<0.001	<0.001
Extracellular matrix binding [GO:0050840]	13	2.10 (1.52 to 2.89)	<0.001	<0.001

The terms associated with significantly different protein expression between the two tissue types before the correction for multiple comparisons are shown in the table.

^a^*P* value corrected for multiple comparisons.

The collagen composition of cNFs appeared more diverse than that of the skin, as the expression level of many collagens was higher in cNFs compared to the skin while only a few types of collagens were expressed at lower levels in cNFs than in the overlying skin (Table 2). The collagens expressed at higher levels in cNFs compared to skin included, for example, types XI, XV, XVIII, and XXVIII. Only collagens type I alpha 1 and 2 (*COL1A1* and *COL1A2*) and type XVII alpha 1 (*COL17A1*) showed statistically significantly lower expression in cNFs compared to the skin (Table 2). Immunohistochemistry confirmed the abundant expression of collagens XV and XVIII in cNFs (Fig. 2).

**Table 2:** A comparison of the expression levels of collagens in cutaneous neurofibromas vs. the overlying skin.

Gene	UniProt ID	Estimate (95% CI)	*P*_*corrected*_^a^
*COL1A1*	P02452	0.41 (0.29 to 0.59)	**0.002**
*COL1A2*	P08123	0.49 (0.38 to 0.62)	**<0.001**
*COL3A1*	P02461	0.77 (0.56 to 1.06)	1.000
*COL4A1*	P02462	2.21 (1.36 to 3.57)	0.078
*COL4A2*	P08572	2.54 (1.77 to 3.65)	**0.001**
*COL5A1*	P20908	1.15 (0.98 to 1.34)	1.000
*COL5A2*	P05997	1.45 (1.05 to 2.02)	0.762
*COL5A3*	P25940	2.19 (1.65 to 2.90)	**0.001**
*COL6A1*	P12109	1.57 (1.00 to 2.46)	1.000
*COL6A2*	P12110	1.72 (1.09 to 2.72)	0.617
*COL6A3*	P12111	1.03 (0.29 to 3.72)	1.000
*COL6A5*	A8TX70	1.91 (0.95 to 3.84)	1.000
*COL6A6*	A6NMZ7	0.74 (0.40 to 1.36)	1.000
*COL7A1*	Q02388	0.08 (0.01 to 0.50)	0.193
*COL11A1*	P12107	356 (19.4 to 6543)	**0.008**
*COL12A1*	Q99715	0.72 (0.53 to 0.97)	0.762
*COL14A1*	Q05707	0.72 (0.38 to 1.35)	1.000
*COL15A1*	P39059	1.95 (1.57 to 2.41)	**<0.001**
*COL16A1*	Q07092	2.28 (1.75 to 2.97)	**<0.001**
*COL17A1*	Q9UMD9	0.02 (0.00 to 0.15)	**0.012**
*COL18A1*	P39060	1.63 (1.38 to 1.93)	**<0.001**
*COL21A1*	Q96P44	1.97 (1.23 to 3.16)	0.148
*COL28A1*	Q2UY09	2.10 (1.53 to 2.88)	**0.007**

*P* values indicating statistically significant difference between cutaneous neurofibromas and the skin are highlighted with boldface.

^a^ *P* value corrected for multiple comparisons.

Nevertheless, the expression of proteins related to the GO term for collagen biosynthetic process (GO:0032964) showed a 40% (95% CI 24% to 53%) lower level in cNFs compared to the skin (Table 1). The GO term for extracellular matrix disassembly (GO:0022617) was also associated with a 59% (95% CI 48% to 68%) lower expression in cNFs than in the skin. These findings suggest a lower level of matrix remodeling in cNFs than in the skin despite the abundant extracellular matrix in cNFs. Extracellular matrix disassembly was positively correlated with angiogenesis (*P* < 0.001), cell proliferation (*P* = 0.030), and the positive regulation of MAPK cascade (*P* = 0.007) in cNFs.

### Angiogenesis

Consistent with the relatively low level of extracellular matrix remodeling in cNFs, also the GO terms related to angiogenesis suggested an overall quiescent microenvironment. A total of 51 proteins contributed to the GO term angiogenesis (GO:0001525) with no difference between cNFs and the skin (0.99, 95% CI 0.92 to 1.07, *P* = 1.000). The sensitivity analyses of growing tumors only, and tumors from patient 4 only yielded practically identical estimates. Several GO terms related to angiogenesis were associated with statistically significantly lower protein expression levels in cNFs compared to the skin (Table 3). Endothelial cell migration (GO:0043542) showed a 31% (95% CI 16% to 43%) lower level, and the regulation of angiogenesis (GO:0045765) was associated with a 28% (95% CI 14% to 40%) lower level in cNFs versus the skin (Table 3). The positive regulation of angiogenesis (GO:0045766) was 24% (95% CI 13% to 33%) lower, and the establishment of endothelial barrier (GO:0061028) showed a 15% (95% CI 6% to 23%) lower level in cNFs compared to the skin. Also the GO terms for positive regulation of endothelial cell chemotaxis (GO:2001028), endothelial cell proliferation (GO:0001935), negative (GO:1904706) and positive (GO:1904707) regulation of vascular associated smooth muscle cell proliferation, and positive regulation of blood vessel endothelial cell migration (GO:0043536) were associated with clearly lower levels in cNFs compared to the skin (Table 3). However, these differences were statistically non-significant after applying the correction for multiple comparisons.

**Table 3:** A comparison of Gene Ontology (GO) terms related to angiogenesis in cutaneous neurofibromas vs. the overlying skin.

GO term	Proteins	Estimate (95% CI)	*P*_*plain*_	*P*_corrected_^a^
Positive regulation of endothelial cell chemotaxis [GO:2001028]	5	0.66 (0.50 to 0.88)	0.005	0.135
Endothelial cell migration [GO:0043542]	6	0.69 (0.57 to 0.84)	<0.001	0.006
Regulation of angiogenesis [GO:0045765]	9	0.72 (0.60 to 0.86)	<0.001	0.010
Endothelial cell proliferation [GO:0001935]	5	0.75 (0.61 to 0.91)	0.004	0.122
Positive regulation of angiogenesis [GO:0045766]	32	0.76 (0.67 to 0.87)	<0.001	0.001
Negative regulation of vascular associated smooth muscle cell proliferation [GO:1904706]	9	0.81 (0.70 to 0.93)	0.003	0.075
Positive regulation of vascular associated smooth muscle cell proliferation [GO:1904707]	6	0.81 (0.66 to 1.00)	0.051	1.000
Positive regulation of blood vessel endothelial cell migration [GO:0043536]	10	0.83 (0.73 to 0.96)	0.011	0.297
Establishment of endothelial barrier [GO:0061028]	8	0.85 (0.77 to 0.94)	0.002	0.045
Negative regulation of vascular endothelial growth factor receptor signaling pathway [GO:0030948]	6	1.32 (1.11 to 1.57)	0.002	0.046
Negative regulation of vascular permeability [GO:0043116]	5	1.47 (1.26 to 1.71)	<0.001	<0.001
Cell migration involved in sprouting angiogenesis [GO:0002042]	6	1.64 (1.33 to 2.01)	<0.001	<0.001

The terms associated with significantly different protein expression between the two tissue types before the correction for multiple comparisons are shown in the table.

^a^*P* value corrected for multiple comparisons.

The protein expression related to the negative regulation of vascular endothelial growth factor receptor signaling pathway (GO:0030948) was 32% (95% CI 11% to 57%) higher in cNFs compared to the skin (Table 3). In addition, the negative regulation of vascular permeability (GO:0043116) was 47% (95% CI 26% to 71%) higher. Nevertheless, the cell migration involved in sprouting angiogenesis (GO:0002042) showed a 64% (95% CI 33% to 101%) higher level in cNFs compared to the overlying skin (Table 3). The direction and magnitude of the associations shown in Table 3 persisted in sensitivity analyses (Supplementary Table 2). Angiogenesis was positively yet non-significantly correlated with cell proliferation (*P* = 0.117) and the positive regulation of MAPK cascade (*P* = 0.057) in cNFs.

### Cellular metabolism

The analysis of the GO terms related to cellular metabolism showed lower expression of proteins associated with several processes in cNFs compared to the overlying skin (Table 4). The GO terms for lipid metabolic process (GO:0006629) and carbohydrate metabolic process (GO:0005975) were associated with 26% (95% CI 14% to 37%) and 24% (95% CI 15% to 32%) lower levels, respectively, in cNFs vs. skin (Table 4). The GO term for cellular protein metabolic process (GO:0044267) showed 8.8% (95% CI 1.1% to 17%) higher level in cNFs compared to the skin based on data from 56 different proteins. However, the difference was not statistically significant after correction for multiple comparisons. The sensitivity analyses restricted to growing tumors only, or to tumors from patient 4 only, displayed highly concordant results (Supplementary Table 3).

**Table 4:** A comparison of Gene Ontology (GO) terms related to cellular metabolism in cutaneous neurofibromas vs. the overlying skin.

GO term	Proteins	Estimate (95% CI)	*P*_*corrected*_^a^
Sphingolipid metabolic process [GO:0006665]	5	0.12 (0.05 to 0.28)	<0.001
Nucleotide metabolic process [GO:0009117]	5	0.32 (0.18 to 0.57)	0.018
Regulation of lipid metabolic process [GO:0019216]	11	0.39 (0.26 to 0.59)	0.001
Release of cytochrome c from mitochondria [GO:0001836]	7	0.41 (0.31 to 0.54)	<0.001
Cellular response to glucagon stimulus [GO:0071377]	5	0.44 (0.31 to 0.63)	0.001
One-carbon metabolic process [GO:0006730]	13	0.47 (0.36 to 0.62)	<0.001
Mitochondrial electron transport, cytochrome c to oxygen [GO:0006123]	7	0.57 (0.50 to 0.64)	<0.001
Response to insulin [GO:0032868]	14	0.63 (0.56 to 0.70)	<0.001
Mitochondrial calcium ion transmembrane transport [GO:0006851]	5	0.64 (0.54 to 0.76)	<0.001
Phosphate-containing compound metabolic process [GO:0006796]	6	0.63 (0.55 to 0.72)	<0.001
Triglyceride metabolic process [GO:0006641]	11	0.63 (0.50 to 0.80)	0.012
Mitochondrial electron transport, ubiquinol to cytochrome c [GO:0006122]	9	0.64 (0.55 to 0.74)	<0.001
Mitochondrial respiratory chain complex IV [GO:0005751]	5	0.64 (0.54 to 0.75)	<0.001
Retinoid metabolic process [GO:0001523]	23	0.64 (0.51 to 0.80)	0.010
Mitochondrial respiratory chain complex III [GO:0005750]	8	0.64 (0.55 to 0.74)	<0.001
Mitochondrial proton-transporting ATP synthase complex [GO:0005753]	10	0.67 (0.61 to 0.74)	<0.001
Regulation of insulin secretion [GO:0050796]	6	0.68 (0.56 to 0.83)	0.017
2-oxoglutarate metabolic process [GO:0006103]	8	0.69 (0.63 to 0.75)	<0.001
Mitochondrial ATP synthesis coupled proton transport [GO:0042776]	10	0.69 (0.63 to 0.76)	<0.001
Mitochondrial respiratory chain complex I [GO:0005747]	14	0.70 (0.64 to 0.77)	<0.001
Mitochondrial electron transport, NADH to ubiquinone [GO:0006120]	16	0.70 (0.65 to 0.77)	<0.001
Oxaloacetate metabolic process [GO:0006107]	6	0.71 (0.64 to 0.79)	<0.001
Lipid metabolic process [GO:0006629]	28	0.74 (0.63 to 0.86)	0.018
ATP metabolic process [GO:0046034]	9	0.74 (0.67 to 0.82)	<0.001
Glycosphingolipid metabolic process [GO:0006687]	14	0.74 (0.67 to 0.82)	<0.001
Glutathione metabolic process [GO:0006749]	22	0.75 (0.67 to 0.85)	<0.001
Generation of precursor metabolites and energy [GO:0006091]	10	0.76 (0.68 to 0.85)	<0.001
Carbohydrate metabolic process [GO:0005975]	37	0.76 (0.68 to 0.85)	<0.001
RNA metabolic process [GO:0016070]	22	0.77 (0.73 to 0.82)	<0.001
Regulation of cellular amino acid metabolic process [GO:0006521]	38	0.82 (0.77 to 0.88)	<0.001
Histone mRNA metabolic process [GO:0008334]	7	0.84 (0.77 to 0.90)	0.001
Glycogen metabolic process [GO:0005977]	5	1.34 (1.18 to 1.53)	0.002
Hyaluronan metabolic process [GO:0030212]	5	1.47 (1.27 to 1.70)	<0.001
Aspartate family amino acid metabolic process [GO:0009066]	5	3.48 (1.82 to 6.62)	0.019

Only terms associated with significantly different protein expression between the two tissue types are shown in the table.

^a^*P* value corrected for multiple comparisons.

Angiogenesis was positively correlated with lipid metabolic process in cNFs (*P* = 0.014) but not with carbohydrate (*P* = 0.153) nor protein (*P* = 0.599) metabolism. Cell proliferation showed a positive but non-significant correlation with lipid (*P* = 0.235), carbohydrate (*P* = 0.137) and protein metabolism (*P* = 0.127) in cNFs. This was in contrast to the skin where cell proliferation displayed a significant positive correlation with carbohydrate metabolism (*P* = 0.024). The positive regulation of MAPK cascade was associated with carbohydrate metabolism in cNFs (*P* = 0.025) but not in the skin (*P* = 0.974). There were no significant associations between the positive regulation of MAPK cascade and lipid or protein metabolism in cNFs or in the skin.

## Discussion

The present proteomics comparison of cNFs versus the overlying skin suggests that cNFs are relatively quiescent in terms of extracellular matrix remodeling, angiogenesis and cellular metabolism. In addition, proteins associated with cell proliferation showed lower expression in cNFs than in the skin, which was confirmed immunohistochemically (Fig. 2) and is in concordance with prior literature [10]. The results are consistent with the invariably benign nature and limited growth potential of cNFs, and the constant renewal of epidermis in the skin. Hierarchical clustering of the cNF and skin samples failed to separate cNFs and the skin in most sample donors (Fig. 1). These results suggest that, in contrast to many other tumor types, the pharmacological treatment of cNFs cannot rely on overall differences between the tumor tissue and the surrounding tissue, such as metabolic activity or cell proliferation. Instead, the therapies should exploit cNF-specific characteristics, such as the *NF1* inactivation and the resulting changes in cellular signaling.

Neurofibromas are well known for their abundant extracellular matrix [28, 29]. Recently, Jiang and co-workers performed a mass spectrometry comparison of developing pNFs versus wild-type dorsal root ganglia in a murine model of pNF [47]. They reported pNF-associated increases in multiple GO terms related to extracellular matrix components, similar to the present comparison between human cNFs and the skin. Jiang et al. also found that when ex vivo cultures of dorsal root ganglia harboring developing pNFs were treated with a mitogen-activated protein kinase kinase (MEK) inhibitor, the expression of *COL6A2*, *COL14A1*, *COL15A1*, *COL18A1*, and *COL28A1* decreased [47]. Interestingly, in the present study, three of these collagens, i.e., *COL15A1*, *COL18A1*, and *COL28A1*, showed higher expression in cNFs compared to the skin (Table 2; Fig. 2). These results suggest similarities between human cNFs and murine pNFs, and call for attention towards the effects of MEK inhibition on extracellular matrix in cNFs.

In an analysis of the matrisome of human cNFs using single-cell RNA sequencing, Brosseau et al. highlighted the low expression of type I (*COL1A1* and *COL1A2*) and type XI (*COL11A1*) collagens in cNFs, whereas type VI collagen (*COL6A1*, *COL6A2*, *COL6A3*) was abundantly expressed by neurofibroma fibroblasts [35]. The high expression of type VI collagen in cNFs has been established also earlier [34, 48]. In the present study, we observed lower expression of type I collagen in cNFs compared to the overlying skin (Table 2). However, we observed markedly higher expression of type XI collagen in cNFs compared to the skin, which is in contrast to the findings of Brosseau and co-workers [35]. While some collagen VI chains seemed to be expressed at higher levels in cNFs than in the skin in the present study, these differences were not statistically significant. Single-cell RNA sequencing focuses on ongoing cellular gene expression while mass spectrometry also highlights previously deposited extracellular matrix components, and the seemingly discordant findings may suggest alterations in the collagens produced during the early and later phases of cNF development. Consistent with the known differences between cNF and pNF growth, another single-cell RNA sequencing analysis of cNFs and pNFs from a single individual showed higher proportions of Schwann cells and endothelial cells as well as more angiogenic features in pNFs than in cNFs [49]. This is in accordance with the present results showing relatively low levels of angiogenesis in cNFs. Interestingly, we observed higher expression of collagens type XV and XVIII in cNFs versus the skin (Table 2; Fig. 2). The restin and endostatin fragments of these two collagens, respectively, are known to exert anti-angiogenic functions [50, 51].

The cNFs analyzed in the present study were removed because they caused discomfort to the patient. Thus, the tumors had not been longitudinally assessed for growth prior to tumor excision. We therefore cannot definitely determine whether the tumors had ceased their growth or were still growing, yet we aimed to estimate this based on the patients’ reports. Restricting the analyses to presumably growing tumors yielded results highly concordant with the overall analysis, indicating that even growing cNFs are relatively quiescent compared with the overlying skin. The expression of proteins related to the GO term for cell population proliferation was closer to the skin in the presumably growing tumors than in all tumors, which seems plausible. Coulpier et al. have described initiation, progression and stabilization phases of cNF development based on results from a murine model, and the progression phase is characterized by high proliferation and MAPK activation of the tumorigenic Schwann cells [10]. Concordantly, we observed positive correlations between extracellular matrix disassembly, angiogenesis, cell proliferation and the positive regulation of the MAPK cascade in cNFs. Extracellular matrix disassembly and angiogenesis can also be hypothesized to represent processes active in growing tumors, and the correlations suggest that these processes are downregulated upon the cessation of tumor growth. Importantly, relatively low expression of angiogenesis-related proteins in a non-growing cNF does not preclude the existence of a high density of previously formed vessels.

The present data rely on the success of the separation of cNF from the overlying skin. The two tissues were dissected macroscopically and the separation may therefore be imperfect. However, the higher expression of the Schwann cell marker S100B in cNFs than in the skin, the very low expression of keratins and type XVII collagen in the cNF samples, and the clear differences in specific proteins and GO terms between the two tissue types speak in favor of a highly successful separation of the tumor tissue and the skin. The remaining expression of keratins in cNFs can be explained by the presence of follicular structures present in all cNFs [52]. Naturally, any inferences arising from the present data require future validation using, e.g., immunohistochemistry, as the scope of the present study allowed the immunohistochemical validation of a few markers only (Fig. 2).

Despite being always benign, cNFs represent a major medical problem among individuals with NF1 due to the associated disfigurement, pain and itching, and reduced quality of life. Topically administered treatments for cNFs are of interest due to a presumably better tolerance than of systemic treatments [13, 16]. For the development of compounds and formulations that act optimally and specifically in the cNF tumor tissue, the skin overlying a cNF is a highly relevant point of comparison. We therefore believe that the present dataset can aid the development of pharmacological therapies for cNFs. As a proteomics resource on cNFs, these data complement the previous cNF gene expression datasets [32, 35, 49]. To our knowledge, the present results are the first to show the expression of several proteins, such as collagens type XV and XVIII, in human neurofibromas. The cNFs represent an interesting case of tumor biology, since they are a highly penetrant yet benign consequence of germline *NF1* haploinsufficiency. Thus, the present dataset not only contributes to our knowledge of cNFs but it can also aid in understanding tumor biology more generally.

Taken together, the present findings suggest that human cNFs are overall rather similar to the overlying skin, and many processes often associated with tumors show lower expression levels in cNFs compared to the skin. It is essential to consider the high degree of similarity between the tumor tissue and the overlying skin in the development of pharmacological therapies for cNFs.

## Supplementary information

Supplementary Tables 1-3

## Data Availability

The mass spectrometry dataset is available at 10.7303/syn52943214.
