# Supplementary material for: Mass spectrometric insights into the protein composition of human cutaneous neurofibromas: comparison of neurofibromas with the overlying skin
Source: Br J Cancer. 2025 May 20;133(3):286–94. doi: 10.1038/s41416-025-03055-9 (PMC12322300; doi:10.1038/s41416-025-03055-9)
Supplement: Supplementary file 1 — Supplementary Tables 1-3 [file 41416_2025_3055_MOESM1_ESM.docx]

**Supplementary Tables 1–3 for**

**Mass spectrometric insights into the protein composition of human cutaneous neurofibromas: comparison of neurofibromas with the overlying skin**

by Roope A. Kallionpää, Eija Martikkala, Pekka Haapaniemi, Sanna-Maria Karppinen, Pilvi Riihilä, Anne Rokka, Ilmo Leivo, Taina Pihlajaniemi, Sirkku Peltonen, Juha Peltonen

| **Supplementary Table 1.** Sensitivity analysis of the extracellular matrix-related Gene Ontology (GO) terms included in the main manuscript Table 1. The comparison of cutaneous neurofibromas vs. the overlying skin is shown for growing tumors only, and for tumors from patient 4 only. | | | | | | | |
| --- | --- | --- | --- | --- | --- | --- | --- |
|  |  | **Primary analysis (Table 1)** | | **Sensitivity analysis: only growing tumors** | | **Sensitivity analysis: only tumors from patient 4** | |
| **GO term** | **Proteins** | **Estimate (95% CI)** | ***P*** | **Estimate (95% CI)** | ***P*** | **Estimate (95% CI)** | ***P*** |
| extracellular matrix disassembly [GO:0022617] | 25 | 0.41 (0.32 to 0.52) | <0.001 | 0.49 (0.35 to 0.67) | <0.001 | 0.33 (0.19 to 0.57) | <0.001 |
| collagen biosynthetic process [GO:0032964] | 5 | 0.60 (0.47 to 0.76) | <0.001 | 0.58 (0.41 to 0.82) | 0.003 | 0.83 (0.57 to 1.21) | 0.332 |
| extracellular space [GO:0005615] | 454 | 0.69 (0.66 to 0.72) | <0.001 | 0.71 (0.67 to 0.75) | <0.001 | 0.70 (0.65 to 0.76) | <0.001 |
| mitochondrial matrix [GO:0005759] | 92 | 0.76 (0.73 to 0.79) | <0.001 | 0.83 (0.79 to 0.87) | <0.001 | 0.69 (0.65 to 0.73) | <0.001 |
| extracellular region [GO:0005576] | 554 | 0.83 (0.80 to 0.85) | <0.001 | 0.85 (0.81 to 0.88) | <0.001 | 0.86 (0.81 to 0.92) | <0.001 |
| collagen trimer [GO:0005581] | 20 | 0.84 (0.71 to 0.99) | 0.041 | 0.84 (0.67 to 1.05) | 0.135 | 0.92 (0.75 to 1.12) | 0.384 |
| collagen catabolic process [GO:0030574] | 10 | 0.87 (0.76 to 0.99) | 0.040 | 0.84 (0.74 to 0.94) | 0.004 | 0.91 (0.65 to 1.28) | 0.598 |
| collagen binding [GO:0005518] | 31 | 1.13 (1.00 to 1.27) | 0.047 | 1.10 (0.92 to 1.30) | 0.298 | 1.14 (1.02 to 1.29) | 0.024 |
| cell-matrix adhesion [GO:0007160] | 49 | 1.15 (1.01 to 1.31) | 0.036 | 1.09 (0.92 to 1.30) | 0.317 | 1.25 (1.01 to 1.54) | 0.042 |
| extracellular matrix structural constituent [GO:0005201] | 51 | 1.25 (1.11 to 1.41) | <0.001 | 1.22 (1.05 to 1.42) | 0.012 | 1.21 (0.97 to 1.51) | 0.089 |
| basement membrane organization [GO:0071711] | 8 | 2.09 (1.51 to 2.89) | <0.001 | 2.19 (1.43 to 3.36) | <0.001 | 1.60 (1.23 to 2.08) | 0.001 |
| extracellular matrix binding [GO:0050840] | 13 | 2.10 (1.52 to 2.89) | <0.001 | 1.76 (1.22 to 2.53) | 0.003 | 2.74 (1.36 to 5.52) | 0.006 |

| **Supplementary Table 2.** Sensitivity analysis of the angiogenesis-related Gene Ontology (GO) terms included in the main manuscript Table 3. The comparison of cutaneous neurofibromas vs. the overlying skin is shown for growing tumors only, and for tumors from patient 4 only. | | | | | | | |
| --- | --- | --- | --- | --- | --- | --- | --- |
|  |  | **Primary analysis (Table 3)** | | **Sensitivity analysis: only growing tumors** | | **Sensitivity analysis: only tumors from patient 4** | |
| **GO term** | **Proteins** | **Estimate (95% CI)** | ***P*** | **Estimate (95% CI)** | ***P*** | **Estimate (95% CI)** | ***P*** |
| positive regulation of endothelial cell chemotaxis [GO:2001028] | 5 | 0.66 (0.50 to 0.88) | 0.005 | 0.74 (0.55 to 1.00) | 0.057 | 0.58 (0.36 to 0.93) | 0.029 |
| endothelial cell migration [GO:0043542] | 6 | 0.69 (0.57 to 0.84) | <0.001 | 0.67 (0.53 to 0.86) | 0.002 | 0.77 (0.58 to 1.01) | 0.062 |
| regulation of angiogenesis [GO:0045765] | 9 | 0.72 (0.60 to 0.86) | <0.001 | 0.71 (0.55 to 0.91) | 0.008 | 0.79 (0.55 to 1.12) | 0.185 |
| endothelial cell proliferation [GO:0001935] | 5 | 0.75 (0.61 to 0.91) | 0.004 | 0.77 (0.63 to 0.93) | 0.008 | 0.82 (0.62 to 1.08) | 0.154 |
| positive regulation of angiogenesis [GO:0045766] | 32 | 0.76 (0.67 to 0.87) | <0.001 | 0.83 (0.72 to 0.97) | 0.017 | 0.70 (0.54 to 0.90) | 0.007 |
| negative regulation of vascular associated smooth muscle cell proliferation [GO:1904706] | 9 | 0.81 (0.70 to 0.93) | 0.003 | 0.87 (0.73 to 1.02) | 0.092 | 0.78 (0.61 to 0.98) | 0.034 |
| positive regulation of vascular associated smooth muscle cell proliferation [GO:1904707] | 6 | 0.81 (0.66 to 1.00) | 0.051 | 0.91 (0.75 to 1.09) | 0.306 | 0.62 (0.39 to 1.01) | 0.059 |
| positive regulation of blood vessel endothelial cell migration [GO:0043536] | 10 | 0.83 (0.73 to 0.96) | 0.011 | 0.95 (0.80 to 1.12) | 0.509 | 0.76 (0.58 to 0.99) | 0.047 |
| establishment of endothelial barrier [GO:0061028] | 8 | 0.85 (0.77 to 0.94) | 0.002 | 0.90 (0.79 to 1.02) | 0.102 | 0.86 (0.75 to 0.99) | 0.036 |
| negative regulation of vascular endothelial growth factor receptor signaling pathway [GO:0030948] | 6 | 1.32 (1.11 to 1.57) | 0.002 | 1.36 (1.06 to 1.76) | 0.018 | 1.31 (1.08 to 1.59) | 0.008 |
| negative regulation of vascular permeability [GO:0043116] | 5 | 1.47 (1.26 to 1.71) | <0.001 | 1.30 (1.05 to 1.61) | 0.017 | 1.39 (1.15 to 1.69) | 0.002 |
| cell migration involved in sprouting angiogenesis [GO:0002042] | 6 | 1.64 (1.33 to 2.01) | <0.001 | 1.38 (1.06 to 1.80) | 0.018 | 2.16 (1.76 to 2.65) | <0.001 |

| **Supplementary Table 3.** Sensitivity analysis of the cellular metabolism-related Gene Ontology (GO) terms included in the main manuscript Table 4. The comparison of cutaneous neurofibromas vs. the overlying skin is shown for growing tumors only, and for tumors from patient 4 only. | | | | | | | |
| --- | --- | --- | --- | --- | --- | --- | --- |
|  |  | **Primary analysis (Table 4)** | | **Sensitivity analysis: only growing tumors** | | **Sensitivity analysis: only tumors from patient 4** | |
| **GO term** | **Proteins** | **Estimate (95% CI)** | ***P*** | **Estimate (95% CI)** | ***P*** | **Estimate (95% CI)** | ***P*** |
| sphingolipid metabolic process [GO:0006665] | 5 | 0.12 (0.05 to 0.28) | <0.001 | 0.14 (0.05 to 0.46) | 0.002 | 0.07 (0.01 to 0.40) | 0.005 |
| nucleotide metabolic process [GO:0009117] | 5 | 0.32 (0.18 to 0.57) | 0.018 | 0.26 (0.10 to 0.63) | 0.004 | 0.34 (0.10 to 1.16) | 0.090 |
| regulation of lipid metabolic process [GO:0019216] | 11 | 0.39 (0.26 to 0.59) | 0.001 | 0.46 (0.28 to 0.75) | 0.002 | 0.29 (0.12 to 0.72) | 0.009 |
| release of cytochrome c from mitochondria [GO:0001836] | 7 | 0.41 (0.31 to 0.54) | <0.001 | 0.42 (0.28 to 0.62) | <0.001 | 0.38 (0.21 to 0.67) | 0.001 |
| cellular response to glucagon stimulus [GO:0071377] | 5 | 0.44 (0.31 to 0.63) | 0.001 | 0.42 (0.23 to 0.75) | 0.004 | 0.50 (0.33 to 0.74) | 0.001 |
| one-carbon metabolic process [GO:0006730] | 13 | 0.47 (0.36 to 0.62) | <0.001 | 0.63 (0.50 to 0.80) | <0.001 | 0.45 (0.29 to 0.70) | 0.001 |
| mitochondrial electron transport, cytochrome c to oxygen [GO:0006123] | 7 | 0.57 (0.50 to 0.64) | <0.001 | 0.64 (0.56 to 0.73) | <0.001 | 0.53 (0.45 to 0.61) | <0.001 |
| response to insulin [GO:0032868] | 14 | 0.63 (0.56 to 0.70) | <0.001 | 0.67 (0.59 to 0.76) | <0.001 | 0.57 (0.47 to 0.70) | <0.001 |
| mitochondrial calcium ion transmembrane transport [GO:0006851] | 5 | 0.64 (0.54 to 0.76) | <0.001 | 0.71 (0.57 to 0.87) | 0.002 | 0.65 (0.55 to 0.77) | <0.001 |
| phosphate-containing compound metabolic process [GO:0006796] | 6 | 0.63 (0.55 to 0.72) | <0.001 | 0.74 (0.61 to 0.91) | 0.005 | 0.48 (0.38 to 0.60) | <0.001 |
| triglyceride metabolic process [GO:0006641] | 11 | 0.63 (0.50 to 0.80) | 0.012 | 0.62 (0.44 to 0.86) | 0.005 | 0.59 (0.36 to 0.97) | 0.040 |
| mitochondrial electron transport, ubiquinol to cytochrome c [GO:0006122] | 9 | 0.64 (0.55 to 0.74) | <0.001 | 0.77 (0.69 to 0.86) | <0.001 | 0.57 (0.46 to 0.69) | <0.001 |
| mitochondrial respiratory chain complex IV [GO:0005751] | 5 | 0.64 (0.54 to 0.75) | <0.001 | 0.75 (0.66 to 0.86) | <0.001 | 0.66 (0.56 to 0.79) | <0.001 |
| retinoid metabolic process [GO:0001523] | 23 | 0.64 (0.51 to 0.80) | 0.010 | 0.54 (0.38 to 0.77) | 0.001 | 0.88 (0.64 to 1.20) | 0.407 |
| mitochondrial respiratory chain complex III [GO:0005750] | 8 | 0.64 (0.55 to 0.74) | <0.001 | 0.79 (0.70 to 0.89) | <0.001 | 0.58 (0.46 to 0.72) | <0.001 |
| mitochondrial proton-transporting ATP synthase complex [GO:0005753] | 10 | 0.67 (0.61 to 0.74) | <0.001 | 0.78 (0.69 to 0.87) | <0.001 | 0.64 (0.56 to 0.74) | <0.001 |
| regulation of insulin secretion [GO:0050796] | 6 | 0.68 (0.56 to 0.83) | 0.017 | 0.72 (0.57 to 0.92) | 0.010 | 0.63 (0.43 to 0.91) | 0.017 |
| 2-oxoglutarate metabolic process [GO:0006103] | 8 | 0.69 (0.63 to 0.75) | <0.001 | 0.78 (0.70 to 0.88) | <0.001 | 0.58 (0.50 to 0.66) | <0.001 |
| mitochondrial ATP synthesis coupled proton transport [GO:0042776] | 10 | 0.69 (0.63 to 0.76) | <0.001 | 0.80 (0.71 to 0.90) | <0.001 | 0.67 (0.58 to 0.76) | <0.001 |
| mitochondrial respiratory chain complex I [GO:0005747] | 14 | 0.70 (0.64 to 0.77) | <0.001 | 0.75 (0.67 to 0.85) | <0.001 | 0.76 (0.64 to 0.90) | 0.002 |
| mitochondrial electron transport, NADH to ubiquinone [GO:0006120] | 16 | 0.70 (0.65 to 0.77) | <0.001 | 0.76 (0.68 to 0.85) | <0.001 | 0.75 (0.64 to 0.87) | <0.001 |
| oxaloacetate metabolic process [GO:0006107] | 6 | 0.71 (0.64 to 0.79) | <0.001 | 0.82 (0.71 to 0.93) | 0.004 | 0.60 (0.50 to 0.71) | <0.001 |
| lipid metabolic process [GO:0006629] | 28 | 0.74 (0.63 to 0.86) | 0.018 | 0.78 (0.64 to 0.96) | 0.021 | 0.71 (0.51 to 0.99) | 0.043 |
| ATP metabolic process [GO:0046034] | 9 | 0.74 (0.67 to 0.82) | <0.001 | 0.81 (0.71 to 0.92) | 0.002 | 0.66 (0.56 to 0.78) | <0.001 |
| glycosphingolipid metabolic process [GO:0006687] | 14 | 0.74 (0.67 to 0.82) | <0.001 | 0.79 (0.71 to 0.89) | <0.001 | 0.60 (0.48 to 0.74) | <0.001 |
| glutathione metabolic process [GO:0006749] | 22 | 0.75 (0.67 to 0.85) | <0.001 | 0.77 (0.64 to 0.91) | 0.003 | 0.76 (0.67 to 0.85) | <0.001 |
| generation of precursor metabolites and energy [GO:0006091] | 10 | 0.76 (0.68 to 0.85) | <0.001 | 0.89 (0.78 to 1.02) | 0.107 | 0.71 (0.63 to 0.81) | <0.001 |
| carbohydrate metabolic process [GO:0005975] | 37 | 0.76 (0.68 to 0.85) | <0.001 | 0.84 (0.73 to 0.96) | 0.010 | 0.61 (0.47 to 0.78) | <0.001 |
| RNA metabolic process [GO:0016070] | 22 | 0.77 (0.73 to 0.82) | <0.001 | 0.80 (0.74 to 0.87) | <0.001 | 0.71 (0.66 to 0.78) | <0.001 |
| regulation of cellular amino acid metabolic process [GO:0006521] | 38 | 0.82 (0.77 to 0.88) | <0.001 | 0.93 (0.88 to 0.98) | 0.010 | 0.67 (0.58 to 0.78) | <0.001 |
| histone mRNA metabolic process [GO:0008334] | 7 | 0.84 (0.77 to 0.90) | 0.001 | 0.88 (0.80 to 0.96) | 0.004 | 0.68 (0.59 to 0.78) | <0.001 |
| glycogen metabolic process [GO:0005977] | 5 | 1.34 (1.18 to 1.53) | 0.002 | 1.40 (1.16 to 1.70) | 0.001 | 1.25 (1.03 to 1.50) | 0.025 |
| hyaluronan metabolic process [GO:0030212] | 5 | 1.47 (1.27 to 1.70) | <0.001 | 1.48 (1.27 to 1.71) | <0.001 | 1.93 (1.59 to 2.34) | <0.001 |
| aspartate family amino acid metabolic process [GO:0009066] | 5 | 3.48 (1.82 to 6.62) | 0.019 | 4.93 (1.92 to 12.67) | 0.001 | 1.67 (0.61 to 4.55) | 0.318 |
